# A pilot study of brisk walking in sedentary combination antiretroviral treatement (cART)- treated patients: benefit on soluble and cell inflammatory markers

**DOI:** 10.1186/s12879-016-2095-9

**Published:** 2017-01-11

**Authors:** Matteo Bonato, Laura Galli, Laura Passeri, Valeria Longo, Gaspare Pavei, Simona Bossolasco, Cecilia Bertocchi, Massimo Cernuschi, Giuseppe Balconi, Giampiero Merati, Adriano Lazzarin, Antonio La Torre, Paola Cinque

**Affiliations:** 1 Department of Biomedical Sciences for Health, Università degli Studi di Milano, 20133 Milan, Italy; 2 Department of Infectious Diseases, San Raffaele Scientific Institute, Milan, Italy; 3 Department of Pathophysiology and Transplantation, Università degli Studi di Milano, Milan, Italy; 4 Department of Radiology, San Raffaele Scientific Institute, Milan, Italy

**Keywords:** cART, Immune activation, Inflammatory markers, Exercise, Physical activity

## Abstract

**Background:**

Chronic HIV infection is associated with low-level inflammation and increased risk of chronic diseases and mortality. The objective was to assess the effects of moderate intensity exercise on metabolic and inflammatory markers in HIV-infected treated persons.

**Methods:**

This was a pilot study enrolling cART-treated, sedentary persons with metabolic complications in a 12-week protocol, consisting of three sessions per week of 60 min brisk walking with (strength-walk group) or without (walk group) 30 min circuit-training. Assessments at baseline and week 12 (W12) included body morphometrics and total body dual-energy X-ray absorptiometry; lipid and glucose blood profile; plasma level of high sensitivity C-reactive protein (hsCRP), interleukin-6 (IL-6), D-dimer, interleukin-18 (IL-18), soluble CD14, and CD38 and HLA-DR expression on CD4+ and CD8+ T-cells.

**Results:**

Forty-nine patients were included and 35 (71%) completed the program: 21 in the walk and 14 in the strength-walk group. At W12, significant improvements were observed of body mass index, waist and hip circumference, and total cholesterol both overall and in the walk group, and of LDL cholesterol in both training groups. In the whole group, significant reductions were observed in hsCRP, IL-6, D-dimer, IL-18, and of CD8+/CD38+/HLA-DR+ cell frequencies. HsCRP and CD8+/CD38+/HLA-DR+ frequency decreased significantly in both training groups when examined separately whereas IL-6 and D-dimer in the walk group only.

**Conclusions:**

Brisk walking, with or without strength exercise, could improve lipid profile and inflammatory markers in chronic HIV infection.

**Trial registration:**

ACTRN12615001258549, registered 17 November 2015, “retrospectively registered” Web address of trial: http://www.ANZCTR.org.au/ACTRN12615001258549.aspx

**Electronic supplementary material:**

The online version of this article (doi:10.1186/s12879-016-2095-9) contains supplementary material, which is available to authorized users.

## Background

Physical activity has been shown to improve the health and quality of life among people with HIV infection [1]. In the general population, it delays all-cause mortality and reduces the risk of cardiovascular disease (CVD), stroke, type-2 diabetes and some types of cancer [2]. These diseases are associated with chronic inflammation, which is characterized by activation of inflammatory signaling pathways with abnormal production of cytokines and other mediators [3]. Observational studies of large population cohorts have consistently shown an association between physical inactivity and low-grade systemic inflammation and interventional studies a reduction of inflammatory markers following exercise [4].

Chronic inflammation is also a predominant feature of treated human immunodeficiency virus (HIV) infection [5, 6]. Compared to age-matched HIV-negative subjects, persons with chronic HIV infection are at higher risk to develop non-acquired immune deficiency syndrome (AIDS) related chronic diseases [7], and several studies have shown an association between chronic inflammation and higher cardiovascular risk and overall mortality [8, 9].

We hypothesized that, like in the general population, physical exercise could decrease inflammation in HIV infection. We designed a pilot study of moderate physical activity [2], consisting of brisk walking, with or without strength exercise, with the objective to assess its effects on metabolic parameters and inflammatory markers in treated HIV-infected persons. In addition, we assessed measures of fitness outcome and evaluated potential differences in all the measures between type of exercise, i.e., resistance only versus resistance plus strength training, and gender. More in general, we also aimed to assess feasibility of the proposed physical activity approach that could help in the design of larger controlled clinical trials.

## Methods

### Study design

This was a 12-week pilot study, which enrolled sedentary HIV-infected patients receiving combination antiretroviral treatment (cART). Inclusion criteria were: age ≥18 years; cART for ≥6 months; sedentary lifestyle, defined as physical activity for <2 days per week for <20 min per session; either objective evidence of lipodystrophy, as established by the visiting physician (PC, SB) [10], or of at least one of the Adult Treatment Panel III definition criteria of the metabolic syndrome [11]. Exclusion criteria included any disease requiring hospitalization in the 6 weeks before enrollment; medical conditions contraindicating exercise as established by a sport medicine specialist; inability to walk at brisk pace; current substance or alcohol abuse. The study protocol was approved by San Raffaele Hospital Ethical Committee (approved on 03/03/2011, prot. N. 142/11) in accordance with current national and international laws and regulations governing the use of human subjects (Declaration of Helsinki II). Written informed consent was obtained from all study participants. This trial was registered retrospectively at Australian New Zealand Clinical Trials Registry (ACTRN12615001258549). This study has not been registered as a clinical trial before its initiation because, at the time it started in March 2011, it was considered exempt from registration based on the Food and Drug Administration Amendments Act (FDAAA) 801 requirements. According to those requirements, “Trials that do not include drugs, biologics, or devices (such as behavioral interventions) … are generally excluded from the registration (and results submission) requirements of FDAAA 801” (https://clinicaltrials.gov/ct2/manage-recs/fdaaa).

### Participant screening and protocol

Participants were recruited from the outpatient Clinic of the Department of Infectious Diseases of San Raffaele Hospital, upon invitation by the caring physician or through advertisement with posters in the Hospital and online through patients’ associations websites. Potential participants were screened for eligibility by an infectious diseases specialist (SB, PC) and a sport medicine specialist (GM), after performing electrocardiogram at rest and during sub-maximal cycle ergometer test. Patients who met the above inclusion criteria and with no contraindications to exercise were offered to either join the ‘walk’ group, consisting of brisk walking only, or the ‘strength-walk’ group, where each walking session was preceded by a strength exercise session.

Participants trained three times a week for 12 weeks. The walking sessions were performed outdoor on measured tracks in green areas in Milan in four groups of 10–15 subjects. To favour adherence to outdoor training, sessions were planned in the periods March-July, 2011 and 2012 (recruitment started on March 4^th^ 2011; last follow up August 15^th^, 2012). Each walking session consisted of 60 min at an intensity of 65–75% of maximal HR (HR_max_) [12]. Each participant was equipped with a personal HR monitor (Polar FT4, Polar Electro 2011, Kempele, Finland) with an acoustic warning if HR was below or exceeded the predetermined range, which was maintained throughout the study. Mean HR (HR_mean_) was recorded during each session, with values captured every 5 s. Strength exercise was carried out in a gym by circuit training, including crunch, lat machine, chest press, leg press, leg extension, sitting calf. Each exercise was repeated 12 times for three sets at 65% of 1-Repetition Maximum (1RM) Test. The walking session was performed outdoor following the resistance training. Professional coaches (MB, GP, ALT) supervised both strength and aerobic training sessions providing technical instruction, supervision and encouragement. Participants received generic dietary advice by the sport medicine specialist involved in the program (GM), consisting of a total food intake of ≤ 2000 Kcal/day (corresponding to ≤ 8374 Kj), including 50–60% carbohydrates, 15–30% proteins and 20–30% fat. Study variables were assessed at baseline (BL) and at the end (W12) of the program. All the demographic and clinical variables were recorded at BL visit through patient interview and access to clinical records.

### Physical fitness evaluation

#### 6 min walking test (6MWT)

Participants were instructed to walk as fast as possible for six minutes on a 400-m outdoor athletic track [13]. HR_mean_ was recorded during the test, blood lactate concentration was assessed before and 3 min after 6MWT (Lactate ProTM, Arkray KdK, Japan), and the Rating of Perceived Exertion (RPE) [14] before and at the end of 6MWT.

#### Strength measurements

1-RM test assessed the maximal load lifted in one repetition for all the strength exercises performed on weight machines, and the 30-s crunch test the number of crunches performed in 30 s.

### Body composition

Anthropometric variables included weight, body mass index (BMI), waist, hip, and thigh circumference on dominant side. Total and % fat mass, lean mass and body mineral content (BMC) at arms, limbs, trunk and as total body was measured by dual-energy X-ray absorptiometry (DEXA) (Lunar Prodigy, version 8.8, GE Medical Systems, Madison, WI). Superficial and visceral fat was measured by ultrasonography at the periumbilical skin-point [15].

### Laboratory analysis

#### Metabolic parameters

Blood examination for metabolic parameters included fasting total, high-density lipoprotein (HDL) and low-density lipoprotein (LDL) cholesterol, triglycerides, glucose, insulin, glycated haemoglobin (HbA1c). Based on these values, the Homeostatic Model Assessment (HOMA)-I index was also calculated.

#### Other blood examinations

Additional examinations included complete blood count and standard biochemical exams; cluster of differentiation 4 (CD4+) and cluster of differentiation 8 (CD8+) T-cell counts, HIV-1-RNA plasma level (Abbott RealTime HIV-1 assay). The Veterans Aging Cohort Study Risk (VACS) index was also calculated [16].

### Inflammatory markers

#### Soluble markers

Soluble biomarkers were measured in cryopreserved plasma samples, drawn at BL and W12, by commercially available enzyme-linked immunosorbent assays according to manufacturers’ recommendation. These included high-sensitivity C-reactive protein (hsCRP, Catalog Number DCRP00), interleukin-6 (IL-6, Catalog Number D6050) and soluble CD14 (sCD14, Catalog Number DC140) (R&D Systems, Minneapolis, MN), D-dimer (Asserachrom, Diagnostica Stago, Asnieres-Sur-Seine, France), interleukin-18 (IL-18) (Medical and Biological Laboratories, Nagoya, Japan).

#### Flow cytometry for cell-activation markers

T-cell activation was measured on cryopreserved peripheral blood mononuclear cells isolated by Ficoll-Paque gradient from EDTA-anticoagulated whole blood. After thawing and PBS-washing, 3 × 10^5^ cells were stained using phycoerythrin (PE)-conjugated anti-HLA-DR, PE-cyanin red 5.1-conjugated anti-CD38, Alexa Fluor 647-conjugated anti-CD3, fluorescein isothiocyanate-conjugated anti-CD4 or anti-CD8 (BD-Biosciences, San Diego, CA). CD38+ and HLA-DR+ cells were gated from the CD3+/CD4+ or CD3+/CD8+ cells on a 2-dimensional dot plot. Analyses were performed by FACSCalibur with CellQuest software (BD-Biosciences) and results reported as percentages of CD3+/CD4+ and CD3+/CD8+ T-cells expressing both HLA-DR and CD38.

For both soluble and inflammatory markers, samples were analysed in batch at the end of the study and blindly with respect to group assignment.

### Retention in study and adherence

Participants were withdrawn from the study if they missed the exercise sessions for ≥ 2 consecutive weeks (corresponding to 6 sessions). Adherence to the program was defined as the proportion of sessions attended during the 12-week training period and it was calculated only among the participants who completed the study.

### Statistical analysis

Quantitative variables were expressed as median and 25–75% interquartiles (Q1-Q3). The Kolmogorov-Smirnov test was performed to test normality of distribution. Since many of the tested variables were not normally distributed, nonparametric tests were used. Changes between BL and W12 were assessed by Wilcoxon matched-pairs signed rank test, BL values and % change differences between groups by Mann–Whitney test, and correlations of continuous variables by the Spearman’s test. Statistical analysis was performed using Graph Pad Prism Software, version 6.0 for Macintosh (Graph Pad Software, San Diego, CA). Level of significance was set at 0.05.

## Results

### Patient disposition and baseline characteristics

**Fig. 1:**
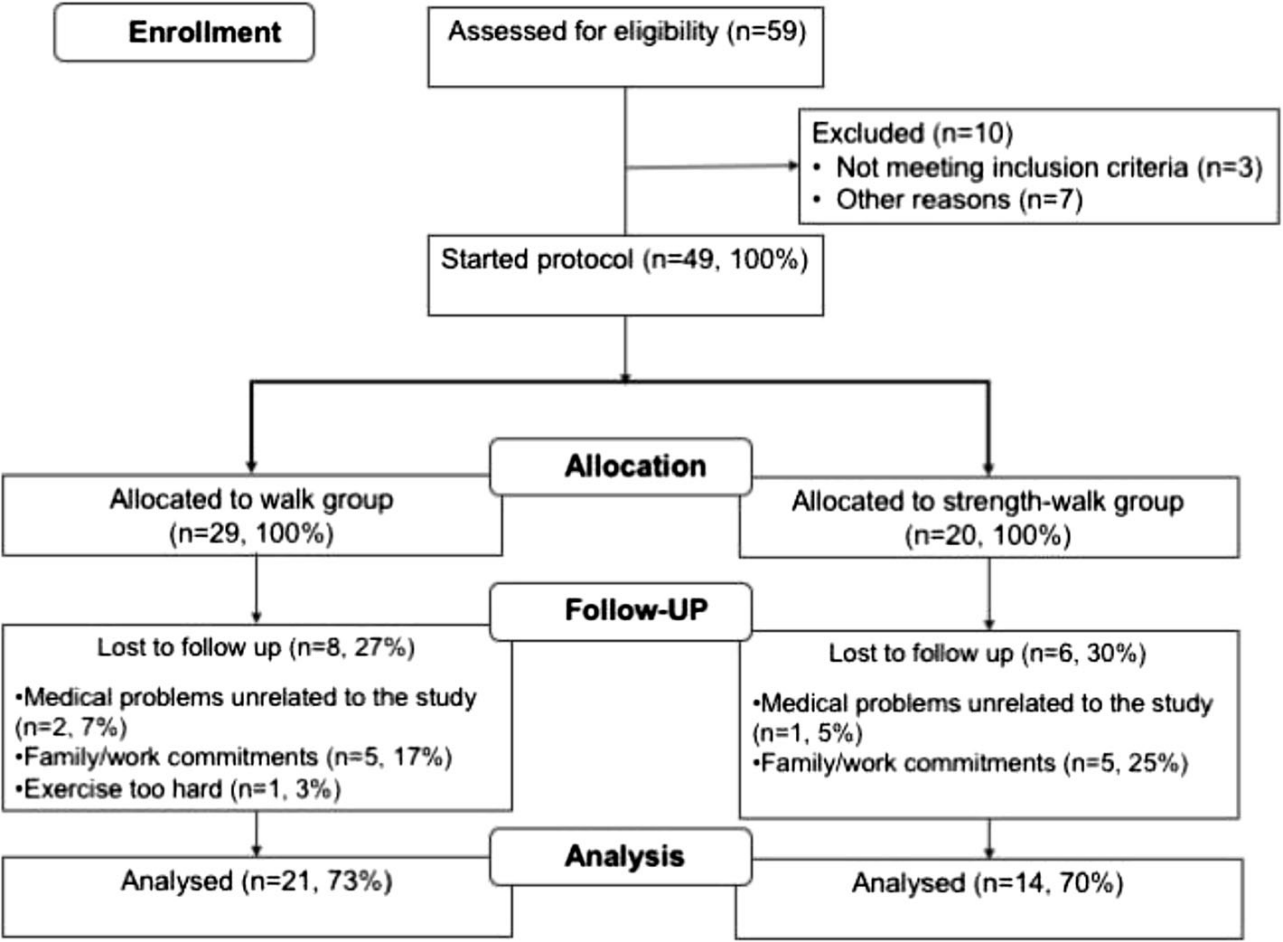
Patient flow diagram

Fifty-nine patients underwent a screening visit and 49 were eligible: 29 joined the walk group and 20 the strength-walk group. Fourteen subjects (29%) dropped out and were not included in the analyses (Fig. 1). Thirty-five subjects were evaluated at W12, including 21 in the walk group and 14 in the strength-walk group.

Of 59 screened patients, 49 were eligible for the study. Non eligibility was due to ambulatory or orthopedic problems, doing exercise > 2 times a week (2 subjects each), not on ART, cardiovascular disease, chemotherapy for lymphoma, renal failure, liver cirrhosis with esophageal varices, severe psychiatric problems (1 subject each). Thirty-five patients completed the study while 14 dropped-out after a median of 5 weeks (range, 2–7). Reasons for drop-out were inability to handle family or work commitments (walk group, *n* = 5; strength-walk group, *n* = 5), medical problems unrelated to the study (walk group, *n* = 2; strength-walk group, *n* = 1), exercise perceived as “too hard” (walk group, *n* = 1).

BL patients’ characteristics are shown in Table 1. Except for gender, there was no difference between the two training subgroups in demographic and clinical variables. Because all women trained in the walking group, post-exercise changes were also analysed according to gender within the walk group.

**Table 1:** Baseline characteristics of the 35 participants who completed the study

	All (*n* = 35)	Walk (*n* = 21)	Strength-Walk (*n* = 14)
Demographic and general characteristics			
Male gender^a^	26 (74%)	12 (57%)^a^	14 (100%)^a^
Age (years, median, Q1-Q3)	48 (44–54)	48 (43–54)	49 (44–54)
Caucasian race	35 (100%)	21 (100%)	14 (100%)
BMI (Kg/m^2^)	25.6 (22.2-27.2)	25.3 (21.6-27.5)	25.8 (24.1-26.9)
Risk Group			
Ex-intravenous drug users	8 (23%)	7 (33%)	1 (7%)
Men-having-sex-with-men	21 (57%)	7 (33%)	13 (93%)
Heterosexual infection	6 (17%)	6 (29%)	0
Vertical infection	1 (3%)	1 (5%)	0
HIV infection variables			
Previous AIDS-defining events	9 (25%)	7 (33%)	2 (14%)
Nadir CD4+ T-cells/μL (median, Q1-Q3)	94 (37–197)	80 (34–140)	188 (77–258)
Current CD4+ T-cells/μL (median, Q1-Q3)	577 (406–726)	485 (374–686)	624 (527–740)
VL < 40 c/mL	34 (97%)	21 (100%)	13 (93%)
Hepatitis C virus coinfection	4 (11%)	4 (19%)	0
VACS Index	12 (5–19)	16 (6–24)	9 (0–12)
Smoking and Treatments			
Smokers	8 (23%)	6 (29%)	2 (14%)
2NRTI + Protease inhibitor	14 (40%)	7 (33%)	7 (50%)
2NRTI + NNRTI	11 (31%)	7 (33%)	4 (29%)
Other ART regimens	10 (28%)	7 (33%)	3 (21%)
Beta blockers^b^	6 (17%)	5 (24%)	1 (7%)
Other anti-hypertensive drugs^b^	5 (14%)	2 (10%)	3 (21%)
Statins^b^	10 (29%)	4 (19%)	6 (42%)
Fibrates^b^	2 (6%)	0	2 (14%)
Inclusion Criteria			
Lipodystrophy	35 (100%)	21 (100%)	14 (100%)
≥ 1 metabolic syndrome criterion	32 (91%)	19 (90%)	13 (93%)
Blood triglycerides ≥150 mg/dL	14 (40%)	8 (38%)	6 (42%)
Blood HDL-C ≤40 (m) or ≤50 (w) mg/dL	12 (34%)	9 (43%)	3 (21%)
Blood glucose ≥110 mg/dL	1 (3%)	0	1 (7%)
Waist ≥102 (m) or ≥88 (w) cm	10 (28%)	8 (38%)	2 (14%)
SBP ≥150 or DBP ≥85 mmHg	12 (34%)	7 (33%)	5 (36%)

Values are expressed as number of patients (%) where no otherwise indicated

^a^Walk vs. strength-walk, *p* = 0.005

^b^Chronic treatment, with no changes during the training period or the 6 weeks before training

*VACS* Veterans Ageing Cohort Study, *NRTI* nucleoside reverse transcriptase inhibitors, *NNRTI* non-nucleoside reverse transcriptase inhibitors, *m* men, *w* women, *SBP* systolic blood pressure, *DBP*, diastolic blood pressure

### Physical fitness

#### Performance during the training sessions

Median overall adherence to the sessions was 67% (dropout subjects were not considered). Participants walked a median distance of 122 km in 12 weeks (5040 m each session) at a median exertion of 66% HR_max_ (Table 2). Participants in the strength-walk group walked longer distances than those in the walk group, both in each session and as a total. No different performances were observed between women and men within the walk group (see Additional file 1: Table S1).

**Table 2:** Participants performance during the 12 weeks of training

	All	Walk	Strength-walk
Adherence (%)	67 (58–75)	61 (56–72)	69 (63–79)
Total walked distance (Km)	122 (84–146)	105^c^ (73–139)	136^c^ (121–155)
Total energy expenditure (KJ/Kg)^a^	298.18 (205.31-356.84)	248.84 (173.00-329.84)	344.67 (306.65-392.82)
Walked distance/session (m)	5040 (4380–5500)	4740^d^ (3535–5390)	5330^d^ (4980–5700)
Energy expenditure per session (KJ/Kg)^a^	12.32 (10.10-14.28)	11.23 (8.01-13.77)	13.51 (12.09-15.25)
HR_mean_ (% of HR_max_)^b^	66 (62–75)	66 (58–73)	70 (63–86)

Values as expressed as median (Q1-Q3). W12 values were compared to BL values by the Wilcoxon matched-pairs signed rank test

^a^Calculation was based on the U shape relationship between walking metabolic cost and speed [43]

^b^The median HR_mean_ value was calculated for each participant through all her/his walking sessions, to derive the median HR_mean_ value of all participants

^c^Walk vs. strength-walk group, *p* = 0.029 (Mann–Whitney test)

^d^Walk vs. strength-walk group, *p* = 0.027 (Mann–Whitney test)

No significant differences were observed between the two training groups for adherence, total and per session energy expenditure, and HR_mean_

*HR* heart rate

#### 6MWT

At W12 6MWT, participants walked for a significantly longer distance compared to BL both in the overall sample and in the two subgroups, in parallel with significant increases of HR_mean_, HR_max_ and delta lactate (Table 3). The distance improvement from BL did not differ between subgroups and between women and men (Additional file 2: Table S2). Overall, better distance improvement correlated with higher adherence (*r* = 0.580; *p* = 0.0003) and longer walked distance (*r* = 0.555; *p* = 0.0005) during the 12 weeks of training (Spearman’s correlation.

**Table 3:** Physical fitness values at baseline (BL) and week-12 (W12)

	All			Walk			Strength-Walk		
	BL	W12	p	BL	W12	p	BL	W12	p
6 min Walking Test									
Distance (m)	642 (605–715)	730 (695–830)	<0.0001	620 (590–701)	700 (660–807)	<0.0001	684 (634–724)	792 (714–850)	<0.0001
HR_mean_ (bpm)	119 (107–132)	137 (116–152)	<0.0001	118 (107–135)	128 (116–154)	0.0003	121 (107–133)	138 (116–153)	0.004
HR_mean_ (%HR_max_)	69 (63–75)	73 (63–75)	<0.0001	69 (59–75)	71 (63–83)	<0.0001	69 (63–75)	75 (68–79)	0.0005
Δ[La^−^] (mmol/L)	0.9 (0.2-2.2)	1.9 (0.5-4.1)	n.s.	0.8 (0.0-2.3)	1.2 (0.5-2.6)	n.s.	1.0 (0.6-2.1)	3.3 (0.7-4.5)	n.s.
Δ RPE	1.0 (0–1)	0.5 (0–2)	n.s.	0.5 (0.0-2.0)	0.5 (0.0-0.2)	n.s.	1.0 (0.0-1.0)	0.5 (0.2-2.0)	n.s.

Values are expressed as median (Q1-Q3). W12 values were compared to BL values by the Wilcoxon matched-pairs signed rank test

*HR* heart rate, *Δ[La* ^*−*^ *]* difference in lactate blood concentration between before and after 6MWT, *Δ RPE* difference in Rate of Perceived Exertion between before and after 6MWT

#### 1-RM and 30-s crunch tests

In the strength-walk group, training was followed by significant improvement of performance for all strength exercises (Additional file 3: Table S3).

### Body composition

Significant reductions were observed of weight, BMI and waist and hip circumference in the whole group. Weight, BMI reduction and waist circumference reductions were maintained in the walk group only (Table 4). However, changes of these parameters from BL did not differ between the two training groups.

**Table 4:** Body composition values at baseline (BL) and week-12 (W12)

	All			Walk			Strength-Walk		
	BL	W12	p	BL	W12	p	BL	W12	p
Anthropometrics									
Weight (kg)	75.0 (65.0-79.0)	72.5 (63.3-77.8)	0.0003	74.0 (62.0-78.5)	71.0 (60–77)	0.0001	76.0 (71.0-81.0)	75.0 (71.5-79.5)	n.s.
BMI (kg/m^2^)	25.6 (22.2-27.2)	25.0 (21.5 -26.9)	0.004	25.3 (21.6-27.5)	24.9 (21.2-27.0)	0.001	25.8 (24.1-26.9)	25.0 (23.9-27.0)	n.s.
Waist Circumference (cm)	93 (86–102)	92 (84–99)	0.050	93 (82–102)	92 (83–100)	0.046	94 (88–100)	92 (88–98)	n.s.
Hip Circumference (cm)	95 (91–99)	94 (90–98)	0.040	97 (92–100)	95 (90–100)	n.s.	92 (91–99)	92 (89–97)	n.s.
Leg Circumference (cm)	53 (49–57)	53 (49–56)	n.s.	53 (47–56)	52 (48–55)	n.s.	53 (51–58)	53 (50–57)	n.s.
Waist-to-hip ratio	0.98 (0.92-0.95)	0.98 (0.95-1.03)	n.s.	0.96 (0.90-0.93)	0.96 (0.93-1.00)	n.s.	1.03 (1.01-1.03)	1.02 (1.00-1.05)	n.s.
DEXA									
Total Fat (Kg)	17.74 (12.52-22.85)	17.66 (9.28-22.41)	n.s.	20.73 (10.07-27.79)	21.48 (9.44-28.94)	n.s.	17.58 (16.16-19.19)	17.52 (10.20-18.38)	n.s.
Total Lean (Kg)	50.88 (47.06-58.23)	52.64 (46.38-57.90)	n.s.	49.52 (38.27-57.55)	48.43 (38.19-57.25)	n.s.	58.22 (50.86-58.24)	57.84 (52.66-59.93)	n.s.
Total BMC (Kg)	2.60 (2.38-2 95)	2.59 (2.35-2.93)	n.s.	2.49 (2.01-2.91)	2.55 (2.30-2.91)	n.s.	2.82 (2.51-3.08)	2.85 (2.48-3.02)	n.s.
Total BMD	1.15 (1.06-1.21)	1.15 (1.06-1.22)	n.s.	1.14 (1.05-1.2)	1.13 (1.03-1.21)	n.s.	1.20 (1.13-1.20)	1.19 (1.07-1.23)	n.s.
Total Fat (%)	25.6 (21.5-33.3)	25.8 (16.7-32.2)	n.s.	28.1 (20.1-37.4)	26.9 (16.7-37.2)	n.s.	24.0 (22.3-27.1)	23.2 (16.5-26.4)	n.s.

Values are expressed as median (Q1-Q3). W12 values were compared to BL values by the Wilcoxon matched-pairs signed rank test

*BMI* body mass index, *DEXA* Dual-energy X-ray absorptiometry, *BMC* body mineral content, *BMD* bone mineral density

No significant changes were observed by DEXA of fat and lean mass or BMC, or of superficial, visceral or total fat by ultrasonography (Table 4, Additional file 4: Table S4). Within the walk group, none of the above parameters was significantly reduced when women and men were analysed separately (Additional file 5: Table S5).

### Laboratory examinations

At W12, significant reductions were observed of total and LDL cholesterol values in the whole sample. Both were also decreased in the walk group, and LDL cholesterol in the strength-walk group (Table 5). Changes from BL did not differ between groups. Within the walk group, cholesterol improvement was observed in men but not in women (see Additional file 5: Table S5).

**Table 5:** Laboratory values at baseline (BL) and week-12 (W12)

	All			Walk			Strength-Walk		
	BL	W12	p	BL	W12	p	BL	W12	p
Total Cholesterol (mg/dL)	196 (163–236)	186 (162–215)	0.018	190 (157–229)	180 (151–214)	0.042	217 (174–237)	191 (165–232)	n.s.
HDL-Cholesterol (mg/dL)	46 (34–54)	47 (36–56)	n.s.	45 (34–55)	47 (35–56)	n.s.	46 (33–56)	45 (35–59)	n.s.
LDL-Cholesterol (mg/dL)	124 (106–155)	108 (93–139)	0.004	116 (96–148)	103 (89–138)	0.033	147 (120–164)	121 (106–144)	0.031
Triglycerides (mg/dL)	143 (111–201)	131 (93–200)	n.s.	142 (101–222)	112 (80–205)	n.s.	137 (107–210)	165 (121–283)	n.s.
Glucose (mg/dL)	85 (78–92)	82 (79–93)	n.s.	85 (74–92)	81 (79–92)	n.s.	86 (80–93)	82 (78–93)	n.s.
Insulin (mg/dL)	11.8 (7.9-16.5)	13.0 (8.0-16.9)	n.s.	10.2 (5.8-17.0)	13.8 (6.6-17.2)	n.s.	13.1 (9.9-15.2)	10.3 (8.6-18.8)	n.s.
HOMA-Index	2.8 (1.6-3.4)	2.7 (1.7-4.1)	n.s.	2.3 (0.9-3.7)	2.8 (1.2-4.1)	n.s.	3.0 (2.1-3.3)	2.3 (1.8-5.0)	n.s.
HbA1c (%)	5.3 (5.1-5.8)	5.5 (5.3-5.6)	n.s.	5.2 (5.0-5.7)	5.3 (5.1-5.6)	n.s.	5.5 (5.3-5.9)	5.5 (5.5-6.0)	n.s.
CD4+ T-cells/μL	577 (407–726)	559 (445–715)	n.s.	485 (365–686)	557 (365–684)	n.s.	642 (527–740)	587 (481–796)	n.s.
CD8+ T-cells/μL	821 (652–914)	747 (566–975)	n.s.	766 (581–872)	743 (632–950)	n.s.	916 (783–1349)	747 (561–1090)	n.s.
VACS index	12 (5–19)	12 (0–21)	n.s.	16 (6–24)	17 (3–22)	n.s.	9 (0–12)	10 (0–18)	n.s.

Values are expressed as median (Q1-Q3). W12 values were compared to BL values by the Wilcoxon matched-pairs signed rank test

*HOMA* Homeostasis Model Assessment, *VACS* Veterans Ageing Cohort Study

Among 25 statin-untreated patients, total, LDL, and also HDL cholesterol improved significantly both in the overall sample and in the walk group, and both total and LDL cholesterol were decreased in the strength-walk group (Additional file 6: Table S6).

No significant changes were observed of the other laboratory variables (Table 5, Additional file 4: Table S4).

### Inflammatory markers

Soluble and cell inflammatory markers were examined in a total of 25 and 16 patients, respectively.

**Fig. 2:**
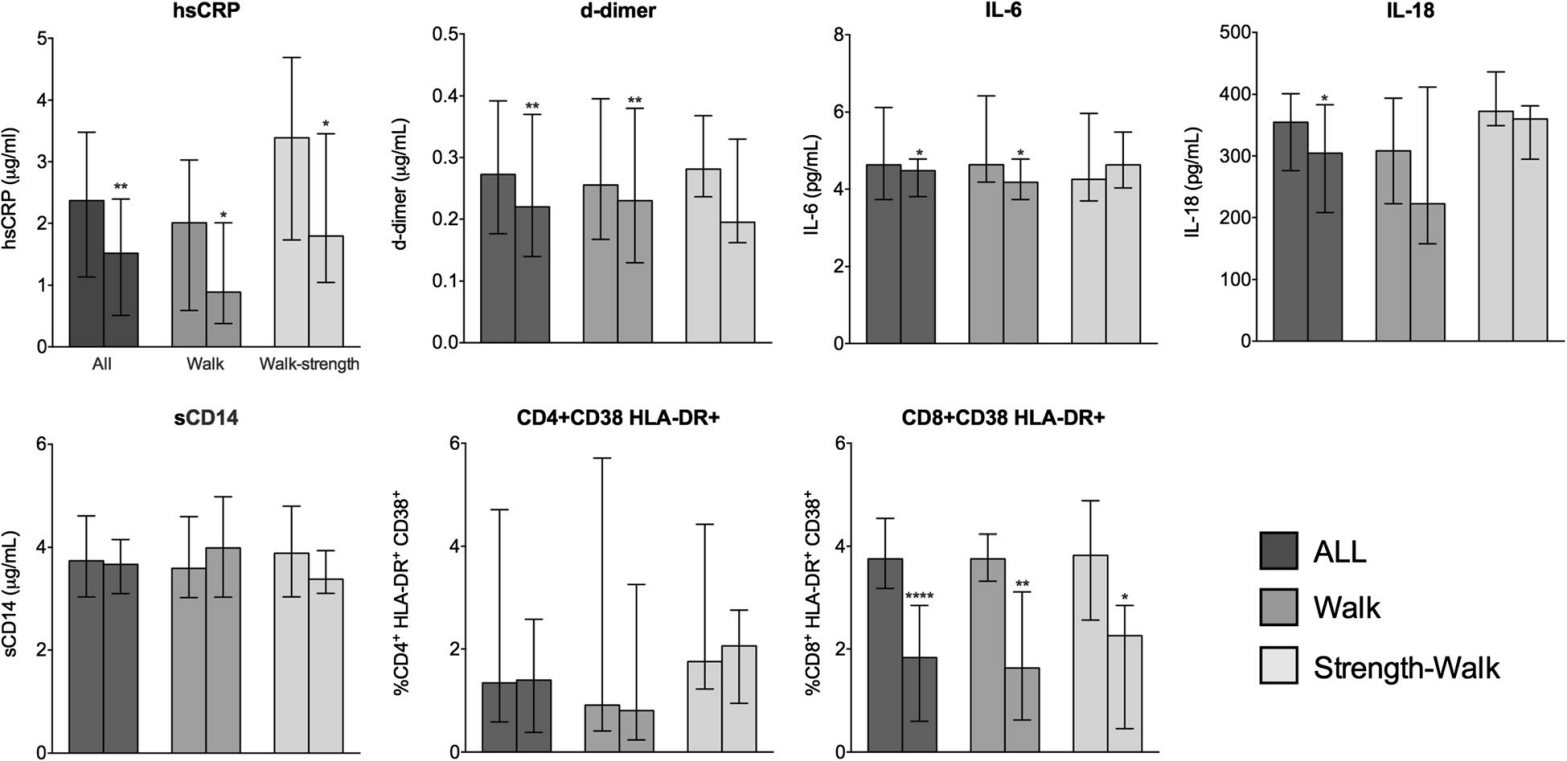
Soluble and cell inflammatory markers at baseline (BL) and week-12 (W12)

Overall, significant reductions were observed of hsCRP, IL-6, D-dimer and IL-18, but not of sCD14, and of CD8+/CD38+/HLA-DR+, but not CD4+/CD38+/HLA-DR+ cell frequencies (Fig. 2). HsCRP and CD8+/CD38+/HLA-DR+ frequency decreased significantly in both training groups, and IL-6 and D-dimer in the walk group only. Subjects with D-dimer concentration above the reference value of 250 ng/mL were 15 of 25 (60%) at BL and 8 of 25 (32%) after exercise (*p* = 0.047, Chi-square test). Subjects with hsCRP above 2 mg/L, i.e., the value considered to confer higher risk for cardiovascular disease in the general population, were 13 of 25 (52%) at BL and 8 of 25 (32%) after 12 week of exercise (*p* = n.s.).

Changes from BL did not differ between training groups. Within the walk group, significant reductions of IL-6 and D-dimer were observed in women and of hsCRP in men (Additional file 7: Table S7).

Twenty-five patients were evaluated for soluble markers (walk, *n* = 15; strength-walk, *n* = 10), and 16 for cell markers (walk, *n* = 10; strength-walk, *n* = 6).

For each group, first and second columns represent values at BL and W12, respectively. Horizontal bars indicate median and Q1-Q3 values (*P < 0.05; **P < 0.01; ****P < 0.0001).

HsCRP, high sensitivity C-reactive protein; IL-6, interleukin-6; IL-8, interleukin-18; sCD14, soluble CD14.

We neither observed significant intercorrelations between changes of inflammatory markers, nor between inflammatory markers and other variables changes.

## Discussion

This pilot study explored the efficacy of a 3-day per week, 12-week program of brisk walking, with or without strength exercise, on metabolic and inflammatory markers in sedentary cART-treated persons with metabolic complications. We observed, in parallel with improvement of physical fitness and of some morphometric measures, substantial improvements of cholesterol profiles and inflammatory markers. Many of the changes were observed in both training groups and, within the walk group, most changes did not differ substantially between women and men.

From a clinical standpoint, a remarkable observation was the general reduction of total cholesterol, the reduction of LDL cholesterol in both training groups, and the increase of HDL among statin-untreated participants. Total, HDL and LDL cholesterol are each independent strong predictors of CVD in the general population and elevated LDL is the primary target for cholesterol-lowering therapy [17, 18]. While high intensity aerobic exercise is followed by favorable cholesterol alterations, the influence of moderate intensity aerobic and of resistance training is not clearly evidence-supported [19]. Only a few studies have examined the effects of exercise on blood lipids in HIV infection, with inconsistent outcomes, likely resulting from large variability of populations and exercise interventions [20–22]. Our findings indicate that moderate exercise may reduce blood cholesterol in HIV infection, supporting exercise interventions including prior to use of cholesterol-lowering drugs [17].

In contrast, we observed no improvement in glucose or insulin level. Functional tests in the general population and HIV-positive patients showed that exercise improves insulin resistance, but often without changes of glucose, insulin or glycate haemoglobin level [22]. Thus, the exercise program followed in this study either had no effect on glucose metabolism or blood static markers did not reflect a possible exercise-induced benefit on glucose control.

Because the HR_mean_ during the training session was set in the so-called fat-burning zone, the observed weight reductions were not unexpected. BMI and waist circumference reductions were less marked in the strength-walk group participants, although they walked longer distances than those in the walking group, likely resulting from increase of muscle mass. Indeed, there was a median increase of lean mass of 1.35 Kg by DEXA examination in the strength-walk group, in contrast from what observed in the walk group, in which no substantial change of lean mass was observed. However, body changes were in general mild, there were no modification of the waist-hip ratio and no significant change in body composition by DEXA or ultrasound examination. Since we prescribed no specific diet, the limited effect of exercise on body fat might reflect an unbalanced caloric intake following exercise. It is also possible that 12 weeks of brisk walking was not sufficient to reduce visceral fat, similar to what observed in obese subjects with type-2 diabetes or dyslipidaemia, likely resulting from a reduced capacity of fat oxidation [23, 24].

cART-controlled chronic HIV infection is associated with increased inflammation and coagulation [5, 6, 25], and higher plasma levels of hsCRP, IL-6 and D-dimer strongly predicted higher overall mortality and cardiovascular events [8, 9]. Beyond cART, a number of interventions are in use, e.g., statins, or proposed, to treat inflammation [26]. However, the anti-inflammatory effect of exercise in HIV infection has been rarely addressed in clinical studies. Reductions of hsCRP, IL-6, TNF-α and IL-18, a cytokine released by adipocytes and other cell types, were observed in a 16-week study of aerobic or resistance training performed at variable intensity [22], but no of IL-6 after 6 weeks of aerobic plus resistance moderate intensity exercise [27].

In HIV-negative subjects, a number of longitudinal studies have demonstrated exercise-induced changes of plasma inflammatory markers. These have largely focused on hsCRP and IL-6, showing more marked reductions in subjects with higher baseline levels, but no or low effect in healthy persons [4, 28]. Scattered studies have shown reductions of plasma IL-18 in old healthy and diabetic subjects [29, 30]. No information is available on the effect of exercise on D-dimer levels, although levels of other coagulation markers may improve following exercise [31].

Thus, our findings extend previous observations in HIV infection by showing an effect of exercise on plasma hsCRP, IL-6 and IL-18 also in patients undergoing moderate intensity exercise. In addition, our findings seem to disclose a beneficial effect of exercise on plasma D-dimer. In contrast to the above markers, we did not observe changes of sCD14, a microbial translocation marker and independent predictor of mortality in chronic HIV infection [32]. Likewise, plasma levels of lipopolysaccharide (LPS) were not reduced by 16 weeks of endurance or strength interventions [33], suggesting no relevant effect of exercise on microbial translocation mechanisms.

Of note, we observed a marked decrease of the frequency of CD8+/CD38+/HLA-DR+ activated T-cells following exercise in both training groups. The effect of physical exercise on T-cell activation is unknown, with only one study showing no change in HLA-DR expression on CD3+ or CD8+ T-cells in HIV-negative elderly following exercise [34]. CD8+ T-cell activation is considered a less strong predictor of mortality and non-AIDS defining events in treated HIV infection than soluble immune activation markers [35, 36]. However, it has been associated with certain non-AIDS comorbidities, such as visceral fat accumulation and subclinical carotid artery disease [37, 38]. Our findings, though preliminary and obtained in a small group of patients, suggest that decreased T-cell activation might contribute to mediate exercise-induced health benefit.

The interpretation of the effect of different exercise programs on inflammatory markers was limited by the small sample size of training groups. However, reductions of hsCRP and CD8+/CD38+/HLA-DR+ cells were observed in both training groups, likely reflecting high sensitivity of these markers to exercise. Also, D-dimer did not improve in the strength-walk group, and there was in this group a non-significant increase of IL-6 concentrations. Similarly, a program of strength exercise alone did not improve plasma hsCRP or IL-6 in a previous study of HIV-infected subjects [22], suggesting that aerobic and resistance exercise might modulate inflammation in different ways [4, 22].

More in general, two main mechanisms have been suggested to mediate the effect of exercise on inflammation. First, the reduction of fat mass following physical activity may promote an anti-inflammatory environment via reduced infiltration of immune cells in the adipose tissue and release of adipokines, including pro-inflammatory cytokines [39]. In addition, contracting skeletal muscle secretes molecules with immunomodulatory effects, including the so-called myokines, most notably IL-6, which mediates metabolic changes during exercise. While single bouts of exercise induce an increase of IL-6 and other cytokines, regular exercise with repeated bouts may induce an anti-inflammatory environment, with lower basal levels of inflammatory markers over time [5]. Compared to the general population, inflammation in HIV infection may be caused or enhanced by specific conditions, including persisting low-level HIV replication, chronic co-infections, and ART-induced altered lipid and metabolic profiles [28], suggesting that additional mechanisms may mediate and perhaps enhance the effects of exercise on inflammation.

From a clinical stand-point, exercise-induced improvement of inflammatory markers may be relevant because persistent low-level inflammation is associated with the presence of chronic diseases, such as CVD, stroke, type-2 diabetes and cancers [2] and increased mortality, both in HIV-positive persons and in the general population [2, 8, 9]. Among the studied inflammatory markers, only hsCRP is currently used in clinical practice to assess the risk of CVD and ischemic events in individuals without manifest disease. Several other markers have the potential to guide and monitor treatment intervention decisions, however their transferability into clinical practice will require efforts in terms of validation of biomarker assays and intervention thresholds and to confirm changes of biomarker levels in the context of clinical trials.

This pilot study also showed that a 12-week exercise program of walking was feasible and associated with acceptable discontinuation rate (28%) and adherence (67%). In addition, none of the participants experienced physical injuries or other medical problems directly related to exercise. Although we did not define ‘a priori’ the feasibility of the proposed intervention, the present study clarified a number of issues defining feasibility, which include the number of eligible participants, the methods and willingness of clinicians to recruit participants; the practicality of the intervention in the given setting and its acceptability to the users, the discontinuation and adherence rates, the availability of data needed and the time needed to collect and analyse data [40].

According to a recent meta-analysis the overall withdrawal rate from exercise interventions was of 24%, with a widely variable range of 0 to 76% [1]. In our study the most frequent reason for study drop-out was the inability of participants to handle with family or work commitments, which often reflects demotivation and is the major reason for not exercising in the general population.

Adherence to the exercise intervention has been reported in only a few studies, with rates of 61–100% [1]. However, criteria for defining adherence vary among studies – e.g., the rate of patients attending at least a given proportion of session rather the actual rate of attended sessions, like in our study. We believe that, in the present study, adherence was favoured by coach supervision during all sessions [1] and, possibly, also by training outdoor during spring-summer. Exercising in natural environments is associated with greater positive engagement compared with exercising indoors [41] and also with greater exercise adherence [42]. However, it seems likely, and also confirmed by our experience, that the effect of outdoor training on adherence may vary depending on weather conditions and thus on the season of the year and geographical area.

This study has some limitations. First, we did not include a non-exercise group as control for marker variations. However, there were no changes of medications, including lipid-lowering drugs, in the weeks before or during the training period, which may have affected study outcomes beyond exercise. Second, assignment to either training protocol was not randomized, but we purposely left the choice of the training activity to participants - the two groups trained in different places at different day times - to favour participation. Although we did not record systematically the reasons for training activity, overall goals differed among participants, with most women wishing to lose weight and most men to improve muscular fitness. This resulted in an unbalanced women distribution between training groups - indeed all the women opted for the walk training program. However, no other relevant differences were observed at baseline between training groups. Also, the relatively small sample size of subgroups, and consequent low statistical power, did not allow drawing firm conclusions on the efficacy of different exercise programs or according to gender or other variables. Finally, dietary intake was not restricted, which might have influenced study outcomes.

Like most longitudinal studies assessing the effects of exercise on different markers [3], this was a relatively short-duration study. Therefore, it will be essential in the future to assess the feasibility and the efficacy of long-term, possibly self-managed, exercise approaches.

## Conclusions

This pilot study suggests that brisk walking may improve cholesterol profile and soluble and cell inflammatory markers in sedentary patients with treated HIV infection and metabolic problems. It provides potentially relevant information for the design of larger controlled studies of moderate physical exercise as treatment of HIV-related chronic immune activation.

## Additional files

Additional file 1: Table S1. Performance of participants during the 12 weeks of training in the walk group divided by gender. Values as expressed as median (Q1-Q3). a. The median HR_mean_ value was calculated for each participant through all his/her walking sessions, to derive the median HR_mean_ value of all participants. HR, heart rate. (DOCX 38 kb)
Additional file 2: Table S2. Physical fitness values by the 6 min Walking Test (6MWT) at baseline (BL) and week-12 (W12) in the walk group divided by gender. Values as expressed as median (Q1-Q3). W12 values were compared to BL values by the Wilcoxon matched-pairs signed rank test. a. At BL, women had higher %HR_mean_ than men (*p* = 0.021, Mann–Whitney test). HR, heart rate; Δ[La^−^], difference in lactate blood concentration between before and after 6MWT; Δ RPE, difference in Rate of Perceived Exertion between before and after 6MWT. (DOCX 48 kb)
Additional file 3: Table S3. 1-Repetition Maximum Test performance at baseline (BL) and week-12 (W12). Values are expressed as median (Q1-Q3). W12 values were compared to BL values by the Wilcoxon matched-pairs signed rank test. HR, heart rate; Δ[La^−^], difference in lactate blood concentration between before and after 6MWT; Δ RPE, difference in Rate of Perceived Exertion between before and after 6MWT. (DOCX 39 kb)
Additional file 4: Table S4. Additional body composition and laboratory values at baseline (BL) and week-12 (W12). Values are expressed as median (Q1-Q3). W12 values were compared to BL values by the Wilcoxon matched-pairs signed rank test. BMI, body mass index; DEXA, Dual-energy X-ray absorptiometry; HOMA, Homeostasis Model Assessment; VACS, Veterans Ageing Cohort Study (DOCX 100 kb)
Additional file 5: Table S5. Body composition and laboratory values at baseline (BL) and week-12 (W12) in the walk group divided by gender. Values as expressed as median (Q1-Q3). W12 values were compared to BL values by the Wilcoxon matched-pairs signed rank test. a. At BL, women had higher % fat in the arm (*p* = 0.015), leg (*p* = 0.034) and as total (*p* = 0.017), lower haemoglobin (*p* = 0.002) and creatinine levels (*p* = 0.008), and higher CD4 cell counts (*p* = 0.009) (Mann–Whitney test). BMI, body mass index; DEXA, Dual-energy X-ray absorptiometry; HOMA, Homeostasis Model Assessment; VACS, Veterans Ageing Cohort Study (DOCX 94 kb)
Additional file 6: Table S6. Values of cholesterol at baseline (BL) and week-12 (W12) in patients untreated with statins. Values are expressed as median (Q1-Q3). W12 values were compared to BL values by the Wilcoxon matched-pairs signed rank test. (DOCX 46 kb)
Additional file 7: Table S7. Values of inflammatory markers at baseline (BL) and week-12 (W12) in the walk group divided by gender. Values as expressed as median (Q1-Q3). W12 values were compared to BL values by the Wilcoxon matched-pairs signed rank test. b. A subset of women (*n* = 3) and men (*n* = 7) were tested for cell activation markers. HsCRP, high sensitivity C-reactive protein; IL-6, interleukin-6; IL-8, interleukin-18; sCD14, soluble CD14. (DOCX 51 kb)

## Abbreviation

1RM: 1 repetition maximum
6MWT: Six minutes walking test
AIDS: Acquired immunodeficiency virus
BL: Baseline
BMC: Body mineral content
BMD: Bone mineral density
BMI: Body mass index
cART: Combination antiretroviral treatment
CD4+: Cluster of differentiation 4
CD8+: Cluster of differentiation 8
CVD: Cardiovascular diseases
DBP: Diastolic blood pressure
DEXA: Dual-energy X-ray absorptiometry
FDAAA: Food and drug administration amendments act
Hb1Ac: Glycated haemoglobin
HDL: High-density lipoprotein
HIV: Human immunodeficiency virus
HOMA: Homeostatic model assessment
HR: Heart rate
HR_max_: Maximal heart rate
HR_mean_: Mean heart rate
hsCRP: High sensitivity c-reactive protein
IL-18: Interleukin 18
IL-6: Interleukin 6
LDL: Low-density lipoprotein
m: Men
NNRTI: Non nucleoside reverse transcriptase inhibitors
NRTI: Nucleoside reverse transcriptase inhibitors
RPE: Rating of perceived exertion
SBP: Systolic blood pressure
sCD14: Soluble CD14
VACS: Veteran aging cohort study risk
VL: Viral load
w: Women
W12: Week 12
Δ RPE: Difference in rate of perceived exertion between before and after 6MWT
Δ[La^−^]: Difference in lactate blood concentration between before and after 6MWT

## References

[1] O’Brien KK, Tynan AM, Nixon SA, Glazier RH (2016). Effectiveness of aerobic exercise for adults living with HIV: systematic review and meta-analysis using the cochrane collaboration protocol. BMC Infect Dis.

[2] Garber CE, Blissmer B, Deschenes MR, Franklin BA, Lamonte MJ, Lee IM, American College of Sports Medicine (2011). American College of Sports Medicine position stand. Quantity and quality of exercise for developing and maintaining cardiorespiratory, musculoskeletal, and neuromotor fitness in apparently healthy adults: guidance for prescribing exercise. Med Sci Sports Exerc.

[3] Hotamisligil GS (2006). Inflammation and metabolic disorders. Nature.

[4] Beavers KM, Brinkley TE, Nicklas BJ (2010). Effect of exercise training on chronic inflammation. Clin Chim Acta.

[5] Lederman MM, Funderburg NT, Sekaly RP, Klatt NR, Hunt PW (2013). Residual immune dysregulation syndrome in treated HIV infection. Adv Immunol.

[6] Deeks SG, Lewin SR, Havlir DV (2013). The end of AIDS: HIV infection as a chronic disease. Lancet.

[7] Guaraldi G, Orlando G, Zona S, Menozzi M, Carli F, Garlassi E (2011). Premature age-related comorbidities among HIV-infected persons compared with the general population. Clin Infect Dis.

[8] Kuller LH, Tracy R, Belloso W, De Wit S, Drummond F, Lane HC (2008). Inflammatory and coagulation biomarkers and mortality in patients with HIV infection. PLoS Med.

[9] Duprez DA, Neuhaus J, Kuller LH, INSIGHT SMART Study Group (2012). Inflammation, coagulation and cardiovascular disease in HIV-infected individuals. PLoS One.

[10] Carr A, Emery S, Law M, Puls R, Lundgren JD, Powderly WG, HIV Lipodystrophy Case Definition Study Group (2003). An objective case definition of lipodystrophy in HIV-infected adults: a case–control study. Lancet.

[11] National Cholesterol Education Program (NCEP) Expert Panel on Detection, Evaluation, and Treatment of High Blood Cholesterol in Adults (Adult Treatment Panel III) (2002). Third Report of the National cholesterol Education Program (NCEP) expert panel on detection, evaluation, and treatment of high blood cholesterol in adults (adult treatment panel III) final report. Circulation.

[12] Tanaka H, Monahan KD, Seals DR (2001). Age-predicted maximal heart rate revisited. J Am Coll Cardiol.

[13] American Toracic Society (ATS) Committee on Proficiency Standards for Clinical Pulmonary Function Laboratories (2002). ATS statement: guidelines for the six-minute walk test. Am J Respir Crit Care Med.

[14] Borg G (1998). Borg’s perceived exertion and pain scales.

[15] Martínez E, Bianchi L, García-Viejo MA, Bru C, Gatell JM (2000). Sonographic assessment of regional fat in HIV-1-infected people. Lancet.

[16] Justice AC, Modur SP, Tate JP, Althoff KN, Jacobson LP, Gebo KA (2013). Predictive accuracy of the veterans aging cohort study index for mortality with HIV infection: a north American cross cohort analysis. J Acquir Immune Defic Syndr.

[17] Stone NJ, Robinson JG, Lichtenstein AH, Bairey Merz CN, Blum CB, Eckel RH (2014). 2013 ACC/AHA guideline on the treatment of blood cholesterol to reduce atherosclerotic cardiovascular risk in adults: a report of the American College of Cardiology/American Heart Association Task Force on practice guidelines. Circulation.

[18] Friis-Møller N, Thiébaut R, Reiss P, Weber R, Monforte AD, De Wit S, DAD study group (2010). Predicting the risk of cardiovascular disease in HIV-infected patients: the data collection on adverse effects of anti-HIV drugs study. Eur J Cardiovasc Prev Rehabil.

[19] Tambalis K, Panagiotakos DB, Kavouras SA, Sidossis LS (2009). Responses of blood lipids to aerobic, resistance, and combined aerobic with resistance exercise training: a systematic review of current evidence. Angiology.

[20] Fillipas S, Cherry CL, Cicuttini F, Smirneos L, Holland AE (2010). The effects of exercise training on metabolic and morphological outcomes for people living with HIV: a systematic review of randomised controlled trials. HIV Clin Trials.

[21] O’Brien K, Nixon S, Tynan AM, Glazier R (2010). Aerobic exercise interventions for adults living with HIV/AIDS. Cochrane Database Syst Rev.

[22] Lindegaard B, Hansen T, Hvid T, van Hall G, Plomgaard P, Ditlevsen S (2008). The effect of strength and endurance training on insulin sensitivity and fat distribution in human immunodeficiency virus-infected patients with lipodystrophy. J Clin Endocrinol Metab.

[23] Ohkawara K, Tanaka S, Miyachi M, Ishikawa-Takata K, Tabata I (2007). A dose–response relation between aerobic exercise and visceral fat reduction: systematic review of clinical trials. Int J Obes (Lond).

[24] Kelley DE, Simoneau JA (1994). Impaired free fatty acid utilization by skeletal muscle in non-insulin-dependent diabetes mellitus. J Clin Invest.

[25] Neuhaus J, Jacobs DR, Baker JV, Calmy A, Duprez D, La Rosa A (2010). Markers of inflammation, coagulation, and renal function are elevated in adults with HIV infection. J Infect Dis.

[26] Klatt NR, Chomont N, Douek DC, Deeks SG (2013). Immune activation and HIV persistence: implications for curative approaches to HIV infection. Immunol Rev.

[27] Dudgeon WD, Jaggers JR, Phillips KD, Durstine JL, Burgess SE, Lyerly GW (2012). Moderate-intensity exercise improves body composition and improves physiological markers of stress in HIV-infected men. ISRN AIDS.

[28] Nimmo MA, Leggate M, Viana JL, King JA (2013). The effect of physical activity on mediators of inflammation. Diabetes Obes Metab.

[29] Kohut ML, McCann DA, Russell DW, Konopka DN, Cunnick JE, Franke WD (2006). Aerobic exercise, but not flexibility/resistance exercise, reduces serum IL-18, CRP, and IL-6 independent of beta-blockers, BMI, and psychosocial factors in older adults. Brain Behav Immun.

[30] Kadoglou NP, Iliadis F, Angelopoulou N, Perrea D, Ampatzidis G, Liapis CD (2007). The anti-inflammatory effects of exercise training in patients with type 2 diabetes mellitus. Eur J Cardiovasc Prev Rehabil.

[31] Lockard MM, Gopinathannair R, Paton CM, Phares DA, Hagberg JM (2007). Exercise training-induced changes in coagulation factors in older adults. Med Sci Sports Exerc.

[32] Sandler NG, Wand H, Roque A, Law M, Nason MC, Nixon DE, INSIGHT SMART Study Group (2011). Plasma levels of soluble CD14 independently predict mortality in HIV infection. J Infect Dis.

[33] Trøseid M, Ditlevsen S, Hvid T, Gerstoft J, Grøndahl T, Pedersen BK, Nielsen SD, Lindegaard B (2014). Reduced trunk fat and triglycerides after strength training are associated with reduced LPS levels in HIV-infected individuals. J Acquir Immune Defic Syndr.

[34] Kapasi ZF, Ouslander JG, Schnelle JF, Kutner M, Fahey JL (2003). Effects of an exercise intervention on immunologic parameters in frail elderly nursing home residents. J Gerontol A Biol Sci Med Sci.

[35] Hunt PW, Sinclair E, Rodriguez B, Shive C, Clagett B, Funderburg N (2014). Gut epithelial barrier dysfunction and innate immune activation predict mortality in treated HIV infection. J Infect Dis.

[36] Tenorio AR, Zheng Y, Bosch RJ, Krishnan S, Rodriguez B, Hunt PW (2014). Soluble markers of inflammation and coagulation but not T-cell activation predict non-AIDS-defining morbid events during suppressive antiretroviral treatment. J Infect Dis.

[37] Guaraldi G, Luzi K, Bellistrì GM, Zona S, da Silva AR D, Bai F (2013). CD8 T-cell activation is associated with lipodystrophy and visceral fat accumulation in antiretroviral therapy-treated virologically suppressed HIV-infected patients. J Acquir Immune Defic Syndr.

[38] Kaplan RC, Sinclair E, Landay AL, Lurain N, Sharrett AR, Gange SJ (2011). T cell activation predicts carotid artery stiffness among HIV-infected women. Atherosclerosis.

[39] Van Gaal LF, Mertens IL, De Block CE (2006). Mechanisms linking obesity with cardiovascular disease. Nature.

[40] Thabane L, Ma J, Chu R, Cheng J, Ismaila A, Rios LP (2010). A tutorial on pilot studies: the what, why and how. BMC Med Res Methodol.

[41] Thompson Coon J, Boddy K, Stein K, Whear R, Barton J, Depledge MH (2011). Does participating in physical activity in outdoor natural environments have a greater effect on physical and mental wellbeing than physical activity indoors? a systematic review. Environ Sci Technol.

[42] Friedenreich CM, MacLaughlin S, Neilson HK, Stanczyk FZ, Yasui Y, Duha A, Lynch BM, Kallal C, Courneya KS (2014). Study design and methods for the breast cancer and exercise trial in Alberta (BETA). BMC Cancer.

[43] Pavei G, Biancardi CM, Minetti AE (2015). Skipping vs running as the bipedal gait of choice in hypogravity. J Appl Physiol.

