# Supplementary material for: A pilot study of brisk walking in sedentary combination antiretroviral treatement (cART)- treated patients: benefit on soluble and cell inflammatory markers
Source: BMC Infect Dis. 2017 Jan 11;17:61. doi: 10.1186/s12879-016-2095-9 (PMC5225655; doi:10.1186/s12879-016-2095-9)
Supplement: Additional file 1: Table S1. — Performance of participants during the 12 weeks of training in the walk group divided by gender. Values as expressed as median (Q1-Q3). a. The median HRmean value was calculated for each participant through all his/her walking sessions, to derive the median HRmean value of all participants. HR, heart rate. (DOCX 38 kb) [file 12879_2016_2095_MOESM1_ESM.docx]

|  |  |  |  |  |  |  |  |  |
| --- | --- | --- | --- | --- | --- | --- | --- | --- |
|  |  |  |  | **Women**  **(n=9)** |  | **Men**  **(n=12)** |  | **p** |
|  |  |  |  |  |  |  |  |  |
| Adherence (%) |  |  |  | 58  (54-65) |  | 66  (61-82) |  | 0.064 |
| Total walked distance (Km) |  |  |  | 76  (67-123) |  | 107  (88-181) |  | n.s. |
| Walked distance/session (m) |  |  |  | 4000  (3445-5330) |  | 4755  (4388-5400) |  | n.s. |
| HR_mean_ (% of HR_max_) ^a^ |  |  |  | 66  (63-77) |  | 63  (56-68) |  | n.s. |
|  |  |  |  |  |  |  |  |  |
