# Supplementary material for: A pilot study of brisk walking in sedentary combination antiretroviral treatement (cART)- treated patients: benefit on soluble and cell inflammatory markers
Source: BMC Infect Dis. 2017 Jan 11;17:61. doi: 10.1186/s12879-016-2095-9 (PMC5225655; doi:10.1186/s12879-016-2095-9)
Supplement: Additional file 2: Table S2. — Physical fitness values by the 6 min Walking Test (6MWT) at baseline (BL) and week-12 (W12) in the walk group divided by gender. Values as expressed as median (Q1-Q3). W12 values were compared to BL values by the Wilcoxon matched-pairs signed rank test. a. At BL, women had higher %HRmean than men (p = 0.021, Mann–Whitney test). HR, heart rate; Δ[La−], difference in lactate blood concentration between before and after 6MWT; Δ RPE, difference in Rate of Perceived Exertion between before and after 6MWT. (DOCX 48 kb) [file 12879_2016_2095_MOESM2_ESM.docx]

|  | **Women (n=9)** | | |  | **Men (n=12)** | | |
| --- | --- | --- | --- | --- | --- | --- | --- |
|  | **BL** | **W12** | **p** |  | **BL** | **W12** | **p** |
|  |  |  |  |  |  |  |  |
| Distance (m) | 620  (577-712) | 697  (614-775) | 0.002 |  | 625  (591-705) | 717  (695-816) | 0.004 |
| HR_mean_ (bpm) | 127  (119-148) | 138  (118-164) | n.s |  | 113  (99-119) | 122  (110-144) | 0.002 |
| HR_mean_ (%HR_max_) | 73 ^a^  (68-85) | 80  (67-91) | n.s |  | 62 ^a^  (59-79) | 69  (67-81) | 0.002 |
| Δ[La^-^](mmol/L) | 0.5  (0.2-3.7) | 1.2  (0.5-3.7) | n.s |  | 0.8  (0.1-1.7) | 3.4  (1.9-6.1) | 0.003 |
| Δ RPE | 0.5  (0.0-1.8) | 0.0  (0.0-0.5) | n.s |  | 0.8  (0.1-2.0) | 1.8  (0.6-2.5) | n.s |
|  |  |  |  |  |  |  |  |
