# Supplementary material for: A pilot study of brisk walking in sedentary combination antiretroviral treatement (cART)- treated patients: benefit on soluble and cell inflammatory markers
Source: BMC Infect Dis. 2017 Jan 11;17:61. doi: 10.1186/s12879-016-2095-9 (PMC5225655; doi:10.1186/s12879-016-2095-9)
Supplement: Additional file 3: Table S3. — 1-Repetition Maximum Test performance at baseline (BL) and week-12 (W12). Values are expressed as median (Q1-Q3). W12 values were compared to BL values by the Wilcoxon matched-pairs signed rank test. HR, heart rate; Δ[La−], difference in lactate blood concentration between before and after 6MWT; Δ RPE, difference in Rate of Perceived Exertion between before and after 6MWT. (DOCX 39 kb) [file 12879_2016_2095_MOESM3_ESM.docx]

|  |  |  | | | |
| --- | --- | --- | --- | --- | --- |
|  |  | **BL** | **W12** | **p** | |
|  |  |  |  | |  |
| Crunch (number) |  | 20  (17-22.0) | 30  (23-34) | | 0.0002 |
| Lat machine (kg) |  | 50  (44-55) | 58  (55-61) | | 0.0002 |
| Chest press (kg) |  | 55  (48-61) | 78  (60-80) | | 0.0007 |
| Leg extension (kg) |  | 40  (39-45) | 50  (45-51) | | 0.014 |
| Leg press (kg) |  | 207  (146-242) | 240  (187-300) | | 0.002 |
| Sitting calf (kg) |  | 53  (43-55) | 70  (59-75) | | 0.0004 |
|  |  |  |  | |  |
