# Supplementary material for: A pilot study of brisk walking in sedentary combination antiretroviral treatement (cART)- treated patients: benefit on soluble and cell inflammatory markers
Source: BMC Infect Dis. 2017 Jan 11;17:61. doi: 10.1186/s12879-016-2095-9 (PMC5225655; doi:10.1186/s12879-016-2095-9)
Supplement: Additional file 4: Table S4. — Additional body composition and laboratory values at baseline (BL) and week-12 (W12). Values are expressed as median (Q1-Q3). W12 values were compared to BL values by the Wilcoxon matched-pairs signed rank test. BMI, body mass index; DEXA, Dual-energy X-ray absorptiometry; HOMA, Homeostasis Model Assessment; VACS, Veterans Ageing Cohort Study (DOCX 100 kb) [file 12879_2016_2095_MOESM4_ESM.docx]

|  |  | | |  | |  | | | | |  | |  | | | | | |  |
| --- | --- | --- | --- | --- | --- | --- | --- | --- | --- | --- | --- | --- | --- | --- | --- | --- | --- | --- | --- |
|  | **All** | | |  | | **Walk** | | | | |  | | **Strength-Walk** | | | | | |  |
|  | **BL** | **W12** | **p** |  | **BL** | | | **W12** | | **p** | |  | | **BL** | | **W12** | | **p** | |
| ***Body Composition*** |  |  |  |  | |  |  | |  | |  | |  | |  | |  | |  |
| ***DEXA*** |  |  |  |  | |  |  | |  | |  | |  | |  | |  | |  |
| Arm Fat (Kg) | 1.74  (126-2.49) | 1.79  (0.96-2.56) | n.s. |  | | 1.78  (1.21-3.00) | 1.86  (1.06-2.86) | | n.s. | |  | | 1.46  (1.27-1.88) | | 1.37  (0.90-1.83) | | n.s. | |  |
| Arm Lean (Kg) | 6.05  (4.89-7.02) | 6.39  (4.97-6.98) | n.s. |  | | 5.87  (3.89-6.31) | 6.02  (4.26-6.43) | | n.s. | |  | | 6.99  (5.86-7.23) | | 7.10  (6.03-7.31) | | n.s. | |  |
| Arm BMC (Kg) | 0.37 (0.31-0.42) | 0.38 (0.30-0.42) | n.s. |  | | 0.36 (0.27-0.42) | 0.38 (0.28-0.42) | | n.s. | |  | | 0.40 (0.33-0.44) | | 0.39 (0.35-0.45) | | n.s. | |  |
| Arm Fat % | 24.2  (15.8-29.7) | 23.8  (11.2-31.0) | n.s. |  | | 28.9  (14.7-38.5) | 25.4  (12.2-37.8) | | n.s. | |  | | 18.9  (15.4-24.1) | | 18.0  (11.2-26.3) | | n.s. | |  |
| Leg Fat (Kg) | 3.48  (2.56-6.45) | 3.39  (2.44-6.89) | n.s. |  | | 3.19  (2.11-7.66) | 3.12  (2.08-9.38) | | n.s. | |  | | 3.78  (3.32-6.05) | | 3.49  (3.25-6.06) | | n.s. | |  |
| Leg Lean (Kg) | 16.54  (14.14-18.94) | 16.55  (14.02-18.84) | n.s. |  | | 16.38  (12.18-17.90) | 15.92  (11.65-18.56) | | n.s. | |  | | 18.28  (14.88-20.69) | | 18.16  (16.54-20.11) | | n.s. | |  |
| Leg BMC (Kg) | 1.00 (0.80-1.12) | 0.98 (0.87-1.13) | n.s. |  | | 0.90 (0.65-1.12) | 0.94 (0.76-1.13) | | n.s. | |  | | 1.09 (0.95-1.16) | | 1.07 (0.93-1.15) | | n.s. | |  |
| Leg Fat % | 18.3  (11.8-26.2) | 17.4  (11.5-25.5) | n.s. |  | | 19.7  (10.2-34.1) | 19.9  (9.6-37.3) | | n.s. | |  | | 17.5  (15.-21.5) | | 16.9  (13.0-21.9) | | n.s. | |  |
| Trunk Fat (g) | 12.43  (8.16-15.50) | 11.94  (6.78-15.83) | n.s. |  | | 13.39  (6.35-16.05) | 15.51  (6.34-16.06) | | n.s. | |  | | 11.77  (9.13-12.99) | | 11.42  (7.58-12.06) | | n.s. | |  |
| Trunk Lean (g) | 25.23  (22.43-27.93) | 26.43  (22.09-27.42) | n.s. |  | | 24.84  (20.69-26.99) | 23.77  (20.44-27.13) | | n.s. | |  | | 26.10  (24.87-28.08) | | 26.96  (26.18-29.04) | | n.s. | |  |
| Trunk BMC (g) | 0.79 (0.68-0.92) | 0.74 (0.67-0.97) | n.s. |  | | 0.73 (0.56-0.91) | 0.73 (0.65-0.98) | | n.s. | |  | | 0.80 (0.71-1.10) | | 0.80 (0.68-0.96) | | n.s. | |  |
| Trunk Fat % | 31.6  (24.9-38.5) | 31.6  (21.4-38.8) | n.s. |  | | 13.4  (6.4-16.2) | 14.2  (5.4-16.0) | | n.s. | |  | | 10.9  (9.1-12.4) | | 11.4  (7.7-12.1) | | n.s. | |  |
| **Ultrasonography** |  |  |  |  | |  |  | |  | |  | |  | |  | |  | |  |
| Superficial Fat (mm) | 17  (12-29) | 18  (12-31) | n.s. |  | | 19  (14-30) | 19  (13-33) | | n.s. | |  | | 13  (8-22) | | 13  (9-18) | | n.s. | |  |
| Visceral Fat (mm) | 59  (50-80) | 65  (55-80) | n.s. |  | | 56  (44-75) | 61  (38-69) | | n.s. | |  | | 65  (54-83) | | 70  (62-90) | | n.s. | |  |
| Total Fat (mm) | 82.0  (67-103) | 85  (76-100) | n.s. |  | | 83  (62-111) | 85  (56-103) | | n.s. | |  | | 82  (66-99) | | 82  (78-100) | | n.s. | |  |
| **Laboratory examinations** |  |  |  |  | |  |  | |  | |  | |  | |  | |  | |  |
| Haemoglobin (mg/dL) | 14.8  (13.7-16.3) | 14.5  (13.0-15.5) | n.s. |  | | 15.1  (13.6-16.3) | 13.0  (14.4-15.5) | | n.s. | |  | | 14.6  (14.1-16.7) | | 14.6  (13.6-15.6) | | n.s. | |  |
| White blood cells (10^-9^/L) | 5.8  (5.1-6.9) | 5.8  (4.9-6.9) | n.s. |  | | 5.5  (4.8-6.4) | 5.4  (4.4-6.3) | | n.s. | |  | | 6.4  (5.4-6.9) | | 6.2  (5.4-7.6) | | n.s. | |  |
| Platelets (10^-9^/L) | 195  (168-254) | 198  (168-248) | n.s. |  | | 195  (168-238) | 199  (160-228) | | n.s. | |  | | 209  (162-279) | | 191  (170-269) | | n.s. | |  |
| Creatinine (mg/dL) | 0.83  (0.76-0.92) | 0.82  (0.71-0.94) | n.s. |  | | 0.82  (0.68-0.92) | 0.8  (0.63-0.86) | | n.s. | |  | | 0.90  (0.81-1.01) | | 0.95  (0.81-0.98) | | n.s. | |  |
| AST (U/l) | 23  (16-29) | 19  (16-33) | n.s. |  | | 23  (15-32) | 20  (14-34) | | n.s. | |  | | 22  (15-27) | | 19  (17-31) | | n.s. | |  |
| ALT (U/l) | 36  (22-53) | 31  (22-44) | n.s. |  | | 33  (22-53) | 30  (20-43) | | n.s. | |  | | 38  (21-57) | | 32  (26-46) | | n.s. | |  |
|  |  | | | | |  | | | | | | | | | | | | |  |
