# Supplementary material for: A pilot study of brisk walking in sedentary combination antiretroviral treatement (cART)- treated patients: benefit on soluble and cell inflammatory markers
Source: BMC Infect Dis. 2017 Jan 11;17:61. doi: 10.1186/s12879-016-2095-9 (PMC5225655; doi:10.1186/s12879-016-2095-9)
Supplement: Additional file 5: Table S5. — Body composition and laboratory values at baseline (BL) and week-12 (W12) in the walk group divided by gender. Values as expressed as median (Q1-Q3). W12 values were compared to BL values by the Wilcoxon matched-pairs signed rank test. a. At BL, women had higher % fat in the arm (p = 0.015), leg (p = 0.034) and as total (p = 0.017), lower haemoglobin (p = 0.002) and creatinine levels (p = 0.008), and higher CD4 cell counts (p = 0.009) (Mann–Whitney test). BMI, body mass index; DEXA, Dual-energy X-ray absorptiometry; HOMA, Homeostasis Model Assessment; VACS, Veterans Ageing Cohort Study (DOCX 94 kb) [file 12879_2016_2095_MOESM5_ESM.docx]

|  | **Women (n=9)** | | |  | **Men (n=12)** | | |
| --- | --- | --- | --- | --- | --- | --- | --- |
|  | **BL** | **W12** | **p** |  | **BL** | **W12** | **p** |
|  |  |  |  |  |  |  |  |
| **Body composition** |  |  |  |  |  |  |  |
| **Anthropometry** |  |  |  |  |  |  |  |
| Weight (kg) | 63.5  (53.5-77.6) | 62  (53-75.5) | n.s |  | 75.0  (68.0-81.0) | 72.0  (63.0-81.0) | 0.004 |
| BMI (kg/m^2^) | 24.7  (21.5-28.7) | 24.0  (21.3-28.5) | n.s |  | 25.3  (21.6-27.5) | 24.9  (21.2-27.0) | n.s |
| Waist circumference (cm) | 93.0  (82.0-106.0) | 98.0  (80.0-100.0) | n.s |  | 93.0  (82.5-102.5) | 92.0  (84.0-99.0) | n.s |
| Hip circumference (cm) | 100.0  (90.0-110) | 100.0  (90.0-107.0) | n.s |  | 97.0  (94.0-98.0) | 95.0  (90.0-98.0) | n.s |
| Leg circumference (cm) | 54.0  (48.0-59.0) | 55.0  (47.0-56.0) | n.s |  | 53.0  (44.0-55.0) | 51.5  (48.3-53.8) | n.s |
| Waist-to-hip ratio | 0.93  (0.90-0.98) | 0.95  (0.93-0.97) | n.s. |  | 0.97  (0.93-1.01) | 0.97  (0.93-1.01) | n.s |
| **DEXA** |  |  |  |  |  |  |  |
| Arm Fat (Kg) | 3.11  (1.45-3.52) | 3.13  (1.41-3.47) | n.s |  | 1.74  (0.55-2.49) | 1.77  (0.58-2.53) | n.s |
| Arm Lean (Kg) | 3.80 (3.39-4.41) | 3.70 (3.47-4.42) | n.s |  | 6.26 (5.85-6.76) | 6.39 (5.77-6.56) | n.s. |
| Arm BMC | 0.26  (0.25-0.32) | 0.27  (0.24-0.31) | n.s |  | 0.40  (0.35-0.43) | 0.41  (0.35-0.43) | n.s. |
| Arm Fat (%) ^a^ | 44.3  (26.0-48.7) | 43.1  (25.4-48.4) | n.s |  | 21.3  (7.6-29.1) | 19.0  (7.3-27.1) | n.s |
| Leg Fat (kg) | 7.66  (2.62-11.47) | 9.38  (2.60-11.19) | n.s |  | 2.82  (1.99-4.41) | 2.70  (1.36-4.48) | n.s |
| Leg Lean (Kg) | 11.80 (10.58-14.00) | 11.42 (10.85-12.89) | n.s |  | 17.26 (16.20-19.18) | 17.42 (15.61-18.90) | n.s. |
| Leg BMC (Kg) | 0.754 (0.64-0.85) | 0.76 (0.63-0.87) | n.s |  | 1.04 (0.82-1.16) | 1.04 (0.92-1.16) | n.s. |
| Leg Fat (%) ^a^ | 32.6  (22.3-49.3) | 37.3  (20.7-46.8) | n.s |  | 11.8  (8.3-21.2) | 11.5  (7.1-21.5) | n.s |
| Trunk Fat (kg) | 15.38  (7.61-18.06) | 15.51  (7.31-18.31) | n.s |  | 13.36  (4.35-15.93) | 15.00  (4.33-15.92) | n.s |
| Trunk Lean (Kg) | 18.44 (17.11-22.98) | 18.66 (17.80-22.11) | n.s. |  | 25.81 (23.07-28.81) | 26.95 (22.28-27.45) | n.s. |
| Trunk BMC (Kg) | 0.73 (0.51-0.94) | 0.70 (0.51-0.90) | n.s. |  | 0.76 (0.62-0.92) | 0.73 (0.68-1.00) | n.s. |
| Trunk Fat (%) | 37.8  (30.0-50.7) | 39.5  (28.8-47.6) | n.s |  | 26.4  (14.1-37.2) | 26.7  (13.6-38.1) | n.s |
| Tot Fat (kg) | 27.99  (12.16-33.59) | 28.94  (11.77-33.94) | n.s |  | 19.24  (8.50-22.85) | 21.14  (8.46-22.41) | n.s |
| Total Lean (Kg) | 37.09 (34.62-43.87) | 37.39 (35.56-43.12) | n.s. |  | 52.26 (49.11-59.73) | 54.11 (48.03-57.98) | n.s. |
| Total BMC (Kg) | 2.39  (1.81-2.55) | 2.37  (1.78-2.53) | n.s. |  | 2.76  (2.29-3.09) | 2.73  (2.40-3.21) | n.s. |
| Tot Fat (%) ^a^ | 37.8  (29.6-48.8) | 35.7  (25.0-47.0) | n.s |  | 23.6  (13.1-30.4) | 26.1  (13.4-31.3) | n.s |
| Total BMD | 1.11 (1.05-1.16) | 1.12 (1.03-1.17) | n.s. |  | 1.16 (1.04-1.29) | 1.16 (1.07-1.25) | n.s. |
| **Ultrasonography** |  |  |  |  |  |  |  |
| Superficial Fat (mm) | 23  (16-37) | 24  (14-37) | n.s |  | 19  (12-24) | 19  (12-31) | n.s |
| Visceral Fat (mm) | 57  (33-67) | 52  (37-69) | n.s |  | 55  (47-102) | 65  (44-74) | n.s |
| Total Fat (mm) | 80  (52-100) | 76  (53-106) | n.s |  | 84  (62-118) | 85  (61-103) | n.s |
| **Laboratory exams** |  |  |  |  |  |  |  |
| Haemoglobin (mg/dL) ^a^ | 13.1  (11.9-14.2) | 13.0  (12.0-14.0) | n.s |  | 16.2  (14.9-16.4) | 15.1  (14.2-15.9) | n.s |
| WBC (10-9/L) | 5.1  (4.6-8.1) | 5.3  (4.4-7.0) | n.s |  | 5.6  (5.1-5.9) | 5.5  (4.3-6.3) | n.s |
| PLT (10-9/L) | 215  (169-294) | 195  (174-271) | n.s |  | 188  (155-230) | 200  (125-220) | n.s |
| Creatinine (mg/dL) ^a^ | 0.67  (0.64-0.77) | 0.65  (0.58-0.69) | n.s |  | 0.84  (0.78-0.92) | 0.81  (0.75-0.94) | n.s |
| AST (U/l) | 20.0  (9.0-22.0) | 19.0  (12.5-31.0) | n.s |  | 29.0  (18.0-51.0) | 22.0  (14.0-37.0) | n.s |
| ALT (U/l) | 25.0  (18.0-36.0) | 23.0  (16.0-36.0) | n.s |  | 47.0  (22.0-66.0) | 42.0  (26.0-70.0) | n.s |
| Total Cholesterol (mg/dL) | 180  (156-287) | 197  (155-239) | n.s |  | 196  (155-227) | 180  (150-201) | n.s |
| HDL (mg/dL) | 51  (41-72) | 55  (44-63) | n.s |  | 37  (32-51) | 37  (31-48) | n.s |
| LDL (mg/dL) | 112  (88-158) | 94  (92-191) | n.s |  | 119  (94-149) | 104  (78-128) | 0.045 |
| Triglycerides (mg/dL) | 95  (59-210) | 102  (73-114) | n.s |  | 158  (123-258) | 148  (87-260) | n.s |
| Glucose (mg/dL) | 81  (71-92) | 79  (75-89) | n.s |  | 85  (78-97) | 88  (80-93) | n.s |
| Insulin (mg/dL) | 8.1  (5.8-16.2) | 13.6  (5.6-23.0) | n.s |  | 13.8  (5.4-17.4) | 14.3  (7.2-16.7) | n.s |
| HOMA index | 1.6  (1.0-3.9) | 2.8  (1.0-4.6) | n.s |  | 2.8  (0.8-3.8) | 2.9  (1.5-3.9) | n.s |
| HbA1c (%) | 5.1  (5.2-5.9) | 5.3  (5.3-5.6) | n.s |  | 5.1  (5.0-5.6) | 5.4  (5.0-5.6) | n.s |
| CD4+ T cells/µL ^a^ | 646  (428-898) | 566  (426-855) | n.s |  | 435  (335-570) | 548  (315-630) | n.s |
| CD8+ T cells/µL | 698  (550-862) | 842  (674-1005) | n.s |  | 776  (615-893) | 696  (502-884) | n.s |
| VACS | 15  (10-28) | 21  (11-22) | n.s |  | 16  (6-18) | 16  (0-18) | n.s |
|  |  |  |  |  |  |  |  |
