# Supplementary material for: A pilot study of brisk walking in sedentary combination antiretroviral treatement (cART)- treated patients: benefit on soluble and cell inflammatory markers
Source: BMC Infect Dis. 2017 Jan 11;17:61. doi: 10.1186/s12879-016-2095-9 (PMC5225655; doi:10.1186/s12879-016-2095-9)
Supplement: Additional file 6: Table S6. — Values of cholesterol at baseline (BL) and week-12 (W12) in patients untreated with statins. Values are expressed as median (Q1-Q3). W12 values were compared to BL values by the Wilcoxon matched-pairs signed rank test. (DOCX 46 kb) [file 12879_2016_2095_MOESM6_ESM.docx]

|  | **All (n=25)** | | |  | **Walk (n=17)** | | |  | **Strength-Walk (n=8)** | | |
| --- | --- | --- | --- | --- | --- | --- | --- | --- | --- | --- | --- |
|  | **BL** | **W12** | **p** |  | **BL** | **W12** | **p** |  | **BL** | **W12** | **p** |
|  |  |  |  |  |  |  |  |  |  |  |  |
| Total Cholesterol (mg/dL) | 202  (178-235) | 193  (173-214) | 0.003 |  | 190  (157-229) | 180  (151-214) | 0.042 |  | 234  (202-236) | 194  (191-232) | 0.010 |
| HDL-C (mg/dL) | 47  (36-55) | 48  (39-56) | 0.016 |  | 47  (36-53) | 48  (37-55) | 0.012 |  | 49  (42-60) | 53  (46-64) | n.s |
| LDL-C (mg/dL) | 147  (110-161) | 121  (93-146) | 0.002 |  | 121  (98-151) | 103  (88-138) | 0.025 |  | 156  (147-172) | 144  (121-150) | 0.002 |
|  |  |  |  |  |  |  |  |  |  |  |  |
|  |  |  |  |  |  |  |  |  |  |  |  |
