# Supplementary material for: A pilot study of brisk walking in sedentary combination antiretroviral treatement (cART)- treated patients: benefit on soluble and cell inflammatory markers
Source: BMC Infect Dis. 2017 Jan 11;17:61. doi: 10.1186/s12879-016-2095-9 (PMC5225655; doi:10.1186/s12879-016-2095-9)
Supplement: Additional file 7: Table S7. — Values of inflammatory markers at baseline (BL) and week-12 (W12) in the walk group divided by gender. Values as expressed as median (Q1-Q3). W12 values were compared to BL values by the Wilcoxon matched-pairs signed rank test. b. A subset of women (n = 3) and men (n = 7) were tested for cell activation markers. HsCRP, high sensitivity C-reactive protein; IL-6, interleukin-6; IL-8, interleukin-18; sCD14, soluble CD14. (DOCX 51 kb) [file 12879_2016_2095_MOESM7_ESM.docx]

|  | **Women Walk (n=6)** | | |  | **Men Walk (n=9)** | | |
| --- | --- | --- | --- | --- | --- | --- | --- |
|  | **BL** | **W12** | **p** |  | **BL** | **W12** | **p** |
| ***Soluble markers*** |  |  |  |  |  |  |  |
| hCRP (µg/mL) | 2.3  (1.0-3.3) | 1.2  (0.5-2.9) | n.s |  | 1.4  (0.4-2.7) | 0.9  (0.4-1.8) | 0.012 |
| IL-6 (pg/mL) | 5.4  (4.7-8.1) | 4.1  (3.4-5.0) | 0.031 |  | 4.5  (3.7-5.5) | 4.2  (3.8-4.6) | n.s |
| D-dimer (µg/mL) | 0.2  (0.2-0.5) | 0.2  (0.1-0.4) | 0.031 |  | 0.3  (0.2-0.4) | 0.2  (0.1-0.4) | n.s |
| sCD14 (µg/mL) | 3.2  (3.0-3.8) | 3.6  (3.0-6.8) | n.s |  | 4.2  (3.1-4.7) | 4.0  (3.2-5.0) | n.s |
| IL-18 (pg/mL) | 335  (306-464) | 228  (187-360) | n.s |  | 273  (164-383) | 215  (149-431) | n.s |
| ***Cell markers*** ^b^ |  |  |  |  |  |  |  |
| CD4+CD38+HLA-DR+ (%) | 0.6  (0.5-0.6) | 0.5  (0.2-2.0) | n.s |  | 1.3  (0.3-6.7) | 1.1  (0.3-5.3) | n.s |
| CD8+CD38+HLA-DR+ (%) | 4.1  (3.5-4.6) | 1.4  (0.3-1.9) | n.s |  | 3.5  (3.1-4.1) | 2.7  (0.7-3.5) | 0.016 |
|  |  |  |  |  |  |  |  |
